# A Convolutional Neural Network Model for Detecting Sellar Floor Destruction of Pituitary Adenoma on Magnetic Resonance Imaging Scans

**DOI:** 10.3389/fnins.2022.900519

**Published:** 2022-07-04

**Authors:** Tianshun Feng, Yi Fang, Zhijie Pei, Ziqi Li, Hongjie Chen, Pengwei Hou, Liangfeng Wei, Renzhi Wang, Shousen Wang

**Affiliations:** ^1^Department of Neurosurgery, Dongfang Affiliated Hospital of Xiamen University, School of Medicine, Xiamen University, Xiamen, China; ^2^Department of Neurosurgery, Fuzhou 900th Hospital, Fuzong Clinical Medical College of Fujian Medical University, Fuzhou, China; ^3^Department of Neurosurgery, Peking Union Medical College Hospital, Chinese Academy of Medical Sciences and Peking Union Medical College, Beijing, China

**Keywords:** pituitary adenoma, deep learning, magnetic resonance imaging, invasion, sellar floor

## Abstract

**Objective:**

Convolutional neural network (CNN) is designed for image classification and recognition with a multi-layer neural network. This study aimed to accurately assess sellar floor invasion (SFI) of pituitary adenoma (PA) using CNN.

**Methods:**

A total of 1413 coronal and sagittal magnetic resonance images were collected from 695 patients with PAs. The enrolled images were divided into the invasive group (*n* = 530) and the non-invasive group (*n* = 883) according to the surgical observation of SFI. Before model training, 100 images were randomly selected for the external testing set. The remaining 1313 cases were randomly divided into the training and validation sets at a ratio of 80:20 for model training. Finally, the testing set was imported to evaluate the model performance.

**Results:**

A CNN model with a 10-layer structure (6-layer convolution and 4-layer fully connected neural network) was constructed. After 1000 epoch of training, the model achieved high accuracy in identifying SFI (97.0 and 94.6% in the training and testing sets, respectively). The testing set presented excellent performance, with a model prediction accuracy of 96%, a sensitivity of 0.964, a specificity of 0.958, and an area under the receptor operator curve (AUC-ROC) value of 0.98. Four images in the testing set were misdiagnosed. Three images were misread with SFI (one with conchal type sphenoid sinus), and one image with a relatively intact sellar floor was not identified with SFI.

**Conclusion:**

This study highlights the potential of the CNN model for the efficient assessment of PA invasion.

## Introduction

Pituitary adenoma (PA) is a common intracranial neoplasm, with a frequency of invasiveness of 35–54% (Lee et al., 2015; Yang and Li, 2019; Principe et al., 2020). PAs with sellar invasion have a high rate of residual tumor and recurrence after surgery and pharmacologic tolerance (Dekkers et al., 2020; Trouillas et al., 2020). According to recent guidelines from the European Society of Endocrinology, temozolomide was considered the first-line chemotherapy for aggressive PAs, radiologically invasive PAs with resistance to conventional treatment (Raverot et al., 2018). Therefore, the accurate radiological diagnosis of PA invasiveness is required to assist the clinician in making therapy strategies and prognosis assessment.

The current preoperative evaluation of sellar invasion is based on radiological characteristics (Bonneville et al., 2020). In previous studies, imaging grading systems of PA invasiveness, such as the Knosp and Hardy grading system, have been widely used to improve the rater reliability and evaluation efficiency (Yip and Aerts, 2016; Mooney et al., 2017b; Bonneville et al., 2020). However, the inter-observer agreement is weak for the Knosp and Hardy grading system (Mooney et al., 2017a). Dichotomizing the full scale was able to address the poor percent agreement of imaging grades. In a recent meta-analysis, PAs with Knosp grade 2, 3A, and 3B presented invasion rates of 30, 61.7, and 81.1%, respectively (Fang et al., 2021). Hence, the invasiveness and non-invasiveness cannot be completely dichotomized according to existing radiographic grades. Although magnetic resonance imaging (MRI) can reveal the sellar structures and tumor characteristics, distinguishing tumor invasion accurately using the naked eye is challenging.

As a branch of machine learning, deep learning has achieved significant improvement in multiple fields of image classification and computer vision, which has also prompted computer reading to become feasible in neuroscience. Machine identification and classification can facilitate faster, accurate, and stable preoperative assessment. Recent advancements in computational power have allowed artificial neural networks to achieve deep architectures due to the graphic processing units and the invention of gradient backward propagation (Wong et al., 2021). These deep neural networks present superior performance than other machine learning techniques and have been progressively applied in clinical practice for intracranial tumors (Akkus et al., 2017; Deepak and Ameer, 2019; Wong et al., 2021). As a deep neural network, convolutional neural networks (CNNs) utilize many learnable convolutional filters to facilitate imaging processing and recognition (Hosny et al., 2018).

This study aimed to construct a CNN model in combination with intraoperative evidence and to assist clinicians in identifying the sellar floor invasion (SFI) of PAs using a contrast-enhanced MRI.

## Materials and Methods

### Patient Cohort

In this study, the keywords including “pituitary adenoma,” “acromegaly,” “Cushing’s disease,” and “hyperprolactinemia” were used to search for electronic medical records from 2015 to 2020. After screening, the clinical data of 695 PAs from 2 medical centers (Fuzhou 900th Hospital and Peking Union Medical College Hospital) were enrolled. Basic clinical data, imaging data, and surgical records were reviewed. This study was approved by the review boards of Fuzhou 900th Hospital and Peking Union Medical College Hospital, and the requirement for informed consent was waived. The inclusion criteria were as follows: (1) patients with clear preoperative imaging data suitable for analysis, (2) transsphenoidal surgery patients with a detailed record of intraoperative invasion, and (3) patients with pathological diagnosis of PAs. Cases with other intracranial tumors, a previous history of surgery or trauma in the sellar region, and artifacts in the imaging were excluded.

### Image Acquisition

**FIGURE 1:**
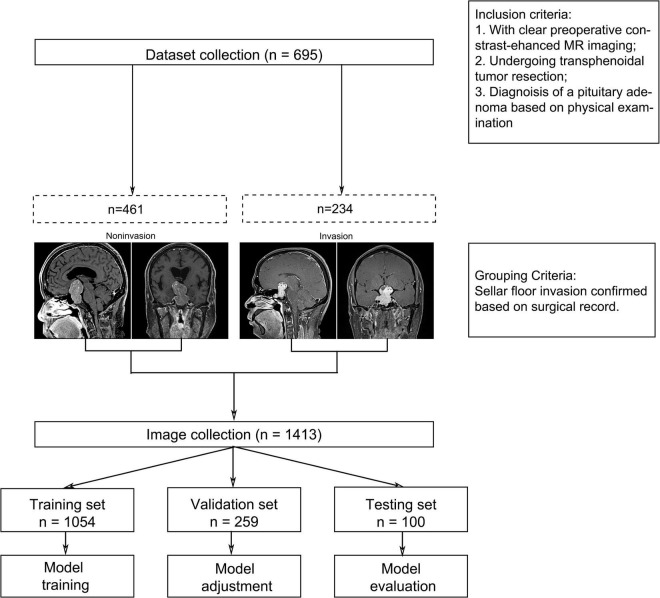
Image data collection process.

All imaging data were collected from contrast-enhanced MRI sequences, including coronal and sagittal scans (Figure 1). In this study, cases were divided into two groups according to surgical evidence. Images of PAs without SFI were collected in the slices with the largest tumor area in both coronal and sagittal scans. The SFI was located by experienced neurosurgeons on the basis of surgical evidence and CT scans, and the images were collected. In the sagittal and coronal MRI images of patients with SFI, except for patients with focal sellar floor destruction, other patients with multiple or diffuse sellar floor destruction were sampled from multilayer sections according to the location of the invasion. These images were then screened by neurosurgeons with more than 20 years of experience in the treatment PAs to remove some MRI images that were blurred or difficult to identify the SFI. After the acquisition, patient information was filtered and eliminated, and only the acquired image was retained.

### Magnetic Resonance Imaging Protocol

All patients in the study were treated with 3.0T MRI machines (Siemens Medical Solutions, Erlangen, Germany, Fuzhou 900th Hospital; Discovery MR 750, GE Healthcare, Peking Union Medical College Hospital). Patients in the two centers for a contrast-enhanced MRI, using the same contrast agent of gadolinium-DTPA (Gd-DTPA) in a small dose. The contrast-enhanced MRI from Peking Union Medical College Hospital acquisition parameters included slice thickness of 3 mm, slice spacing of 0.39 mm, echo time of 9.2 ms, repetition time of 400 ms, and an image size of 512 × 512 × 8 pixels. And, the parameters of contrast-enhanced MRI included scan field of view was 180 × 180 mm with matrix of 320–384 × 240–252, axial slice of 1.0 mm, gap of 1.0 mm, coronal and sagittal section of 1.0 mm with gap of 1.0 mm in Fuzhou 900th Hospital.

### Image Preprocessing

One hundred images were randomly selected from the acquired images, which were not involved in model construction. The remaining 1314 image datasets were randomly grouped at a 80:20 ratio into the training and validation sets to develop a CNN model. The image preprocessing procedure is summarized as follows: (1) All images were converted to 256 × 256 square images using zero padding and image resizing as appropriate (Kim et al., 2019). (2) Images were converted into grayscale with a single channel. (3) Augmentation procedure using horizontal flip and vertical flip and batch normalization were performed for data enhancement.

### Classification With Deep Neural Network

**FIGURE 2:**
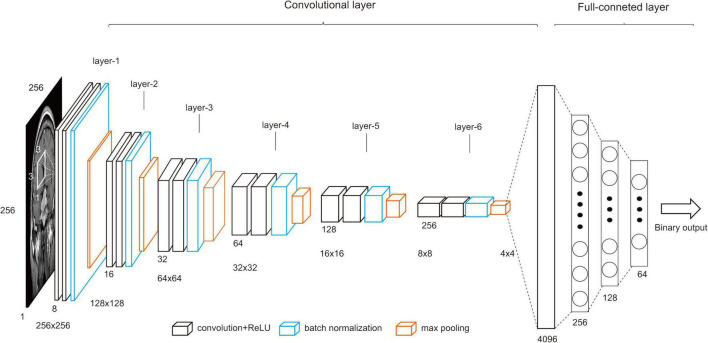
The CNN structure.

The input images to the CNN model were 256 × 256 with single channel. Feature capture was performed using six-layer convolution layers, with 3 × 3 kernel size and zero padding without stride. Each layer included a convolution layer, an activation layer, a BatchNorm2d layer, and a maxpool layer. The maxpool layer used a 2 × 2 matrix. Batch normalization is a standard normal distribution with mean 1 and variance 1. It is used to solve the gradient hour problem and accelerate the convergence rate (parameter: eps = 1e−05, momentum = 0.1, affine = True, track_running_stats = True). Then, 4 × 4 × 256 feature maps were output through six-layer convolution layers, flattening, and connecting to a fully connected neural network of 256 neuronal nodes. The neural network consists of four layers. Finally, the binary classification results were output. In addition to the connecting and output layers, 2 layers of the hidden layer were also included, with 128 and 64 neural nodes, respectively. There were also two dropout layers with a probability of 0.7 and 0.5, respectively. The dropout layer reduces overfitting by randomly omitting partial feature detectors on each training case (Geoffrey et al., 2012). The full connection layer was activated using the ReLU function. A binary cross-entropy function was employed as the loss function, and Adam was used as the optimization function (learning rate = 0.0001). Adam, an algorithm for first-order gradient-based optimization of stochastic objective functions, can adjust different learning rates, is computationally efficient, and has little memory requirements (Kingma and Ba, 2014). The model structure is detailed in Figure 2. The trained model fixes and closes the dropout and batch normalization layers to fit validation set data through the eval function.

### Evaluation

The external testing set was used to assess model generalization capabilities. The 100 testing images that did not participate in model development underwent simple transformation (converting to 256 × 256 square images, single-channel grayscale map, and normalization) into a model with adjusted weights for result testing and evaluation. The eval function that can fix model batch normalization and dropout layer was also used in the testing set. The prediction results were combined with actual labels to establish a confusion matrix. The records included true positive, false positive, true negative, and false negative. The sensitivity, specificity, positive predictive value, negative predictive value, and area under the receptor operator curve (AUC-ROC) were also calculated to assess the model’s predictive and generalization ability.

In addition, the evaluation results of 100 images of all testing set were directly exported to reveal individual cases. The red mark represents the image with evaluation difference. The detailed code is shown in Supplementary Material.

### Statistical Analysis and Software Availability

All imaging data processing and model methods were implemented using PyTorch (version 1.8.1)^1^ and operated in Jupyter Notebook (version 6.4.0).^2^ Several open modules, including Torch.nn, Torch.optim, and DataLoader, can be used to develop a CNN model. Open-source libraries such as Sklearn (version 2.1.0), NumPy (version 1.19.5), and Matplotlib (version 3.4.2) were also used for model performance evaluation and visualization.

The preprocessing of the image datasets depended on the Transforms module in Torchvision library (version 0.9.1). The interface for imaging data loading is the DataLoader module in Torch library (parameters of the training set and validation set: batch size = 4, shuffle = True). The model was developed using Torch.nn module. Optimization was performed using Torch.Adam. CrossEntropyLoss function was used as the loss function. Hiddenlayer library (version 0.3) was used to display the model training results dynamically. The confusion matrix was formed by Confusion_matrix function. AUC-ROC analysis was applied to assess the diagnostic value in identifying invasiveness, which was calculated using the Roc_curve function.

SPSS (version 25) was used for statistical analysis. Categorical variables were summarized as number (percentages) and analyzed by Pearson’s Chi-squared test. Continuous variables were presented as mean ± continuous variables and analyzed by *T*-tests. The significance of differences was accepted at *p* < 0.05.

## Results

In the cohort, 234 (33.7%) cases were intraoperatively confirmed with SFI, and 461 (66.3%) cases had no invasion (Table 1). There were 373 males (53.67%) and 322 females (46.33%), and the incidence of SFI was significantly higher in males than in females (*p* = 0.028). The mean age was 48.6 ± 14.0 years (12–83 years). SFI was not significantly correlated with age (*p* = 0.224). The mean tumor diameter was 28.38 ± 11.00 mm. The tumor diameter in the group with SFI was significantly larger than that in the group without SFI (38.91 ± 10.75 versus 27.20 ± 10.60 mm, *p* < 0.001).

**TABLE 1:** Summary of patient characteristics.

Characteristics *N* = 695	Value
**Age**	
Mean, years	48.6 ± 14.0
Range, years	12.0–83.0
**Sex (%)**	
Female	322 (46.33)
Male	373 (53.67)
**Diameter**	
Mean, mm	28.38 ± 11.00
With SFI, mm	38.91 ± 10.75
Without SFI, mm	27.20 ± 10.60
**Sellar invasion (%)**	
Yes	234 (33.7)
No	461 (66.3)
**Model group (%)**	
Training	1054 (80)
Validation	259 (20)
Testing	100
**Accuracy (%)**	
Training	97.0
Validation	94.6
Testing	96.0

Finally, after sifting through all the images, a total of 1413 images were collected from 695 patients with a PA. Among them, 530 images were collected in cases with SFI, and 883 were collected in the group without SFI. Except for 100 images randomly selected for the external test set (28 in the invasion group and 72 in the non-invasive group; 32 coronal images and 68 sagittal images), the remaining 1313 images were randomly grouped at a ratio of 80:20. Consequently, 1054 images data were enrolled in the training set (400 in the invasion group and 654 in the non-invasive group; 518 coronal images and 536 sagittal images). A total of 259 images were enrolled in the validation set (102 in the invasion group and 157 in the non-invasive group; 135 coronal images and 124 sagittal images). No significant difference in the distribution of coronal and sagittal images has been found between the training set and the validation set (*p* = 0.391). Similarly, we also ascertained no significant difference in the number of invasive images between the training set and the validation set (*p* = 0.671).

**FIGURE 3:**
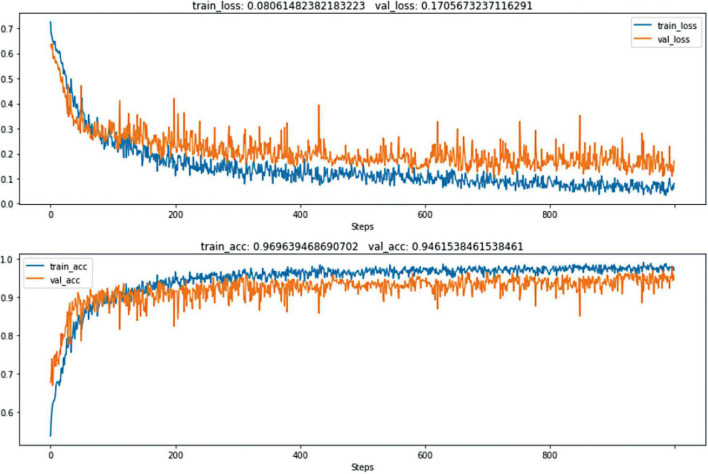
The 1000 epochs of the training process.

**FIGURE 4:**
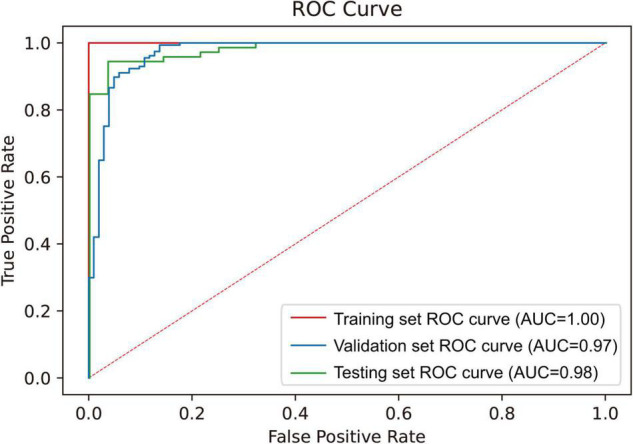
ROC curve of the training set, validation set, and testing set.

With 1000 training epochs, the model presented convergence and achieved an accuracy over 90% (Figure 3). The diagnosis accuracy of SFI was 97.0% in the training set and 94.6% in the validation set. The confusion matrix is shown in Figure 4. The diagnostic accuracy of the testing set was 96%. The sensitivity was 0.964, the specificity was 0.958, the positive predictive value was 0.900, and the negative predictive value was 0.986, the positive likelihood ratio is 22.952. The model had an AUC-ROC value of 0.98. These results showed that the CNN model has excellent diagnostic efficacy to distinguish SFI.

In the four misdiagnosed images of the testing set, three were misread as having SFI and one as having no SFI. The diagnosis results of the testing-set images can be available are shown in Supplementary Material. In the images misread as SFI, one case was diagnosed with conchal type sphenoid sinus, and the other two cases with large PAs presented severe dilatation of the sellar floor. The frequency of conchal type sphenoid sinus was limited in this study, and the model might misunderstand the type of sellar floor. One misread image with SFI had a relatively intact sellar floor, which was not correctly identified by the model.

## Discussion

Imaging is essential for preoperative diagnosis of invasive and aggressive PA. Advanced imaging techniques can clearly reveal the sellar structure to assist clinicians in assessing the invasiveness of PAs (Cao et al., 2013; Bonneville et al., 2020). Furthermore, various imaging grades for PA invasiveness have been reported (Micko et al., 2019; Fang et al., 2021). In the sellar invasion scale of Hardy classification, the percent agreement among all raters improved from 16% (8/50 cases) for the full scale to 64% (32/50 cases) for the dichotomous scale (Mooney et al., 2017b). Although investigators try to develop novel sequences or imaging grades to accurately assess sellar structures, the invasiveness of PAs is difficult to accurately diagnose due to limitations of macroscopic identification, need for advanced imaging facilities, and time constraints (Yoneoka et al., 2008; Cao et al., 2013; Lang et al., 2018).

Machine learning can capture the information of each pixel as meticulously as possible through the algorithm to more accurately identify the details than the naked eye. Machine learning can significantly reduce the cost for centers that unconditionally update more advanced imaging facilities. There have been several tentative applications and investigations in the diagnosis and management of intracranial tumors, including the diagnosis of PAs and tumor characteristics (Deepak and Ameer, 2019; Fan et al., 2020; Wang et al., 2021). A total of 194 PAs with Knosp grade 2–3 was included in the latest radiomic study to identify invasion of the cavernous sinus (ICS) (Niu et al., 2019). This study extracted image data through manual delineation and segmentation, and 2553 image features were analyzed using SVM models to identify ICS. The AUC values of the training and test sets were 0.85 and 0.83, respectively, indicating that the model was reliable to distinguish cases with or without ICS in the Knosp grade. This literature confirms the feasibility of machine learning models in imaging identification and classification of PAs. However, the most critical step in the application of radiomics is the delineation of tumor margins. The quality of delineation will directly affect the results of data analysis. In addition to manual bias, this method has a limited identification range and low efficiency, which is not conducive to clinical application and promotion, so it is more used to study clinical diseases (Yip and Aerts, 2016; Fan et al., 2020).

In the current study, a deep learning-based model was described to accurately classify the invasiveness of PA according to imaging data. With improved algorithms and computer modules, deep neural networks can train and generalize relatively small amounts of data with high sensitivity and specificity. There are few applications of deep learning in the pituitary tumor. Wei et al. (2020) used a deep learning convolution model to discriminate acromegaly (*n* = 1139), Cushing’s disease (*n* = 880), and normal human facial images (*n* = 12,667). The accuracy of the external testing set (*n* = 60) was 91.7%, confirming the reliability of CNN for the diagnosis of PA. Li et al. (2021) recently reported a deep learning network that was constructed using 168 patients with PAs. The model can accurately assess functional and non-functional PAs according to imaging data. In our cohort, 1413 PA images were collected to develop a CNN to diagnose SFI. The accuracy of prediction was 97.0 and 94.6% in the training and validation sets, respectively. The model has high generalization ability. Through the performance evaluation of the 100-case testing set, the prediction accuracy of SFI was 96% with an AUC-ROC value of 0.98. The accuracy of the model for SFI diagnosis is much higher than that of Hardy classification. Therefore, the CNN model might become a valuable tool to identify and correctly diagnose the properties of PAs.

Recognizing the invasiveness of PA through deep learning provides not only an objective and stable basis for surgical strategies and prognostic evaluation, but also a more accurate invasive diagnosis for patients without surgical conditions, especially for aggressive PA requiring drug chemotherapy. The assessment of invasiveness is essential for diagnosing the presence of aggressive PAs that are significantly tolerated by traditional medical and surgical treatment (Lopes, 2017). In the latest guidelines for diagnosing and managing aggressive PAs, temozolomide was considered the first-line pharmacotherapy (Raverot et al., 2018; Luo et al., 2021). Consequently, a higher accuracy diagnosis of sellar invasion is required according to imaging data. The deep machine learning model in this study was confirmed to be stable and effective for diagnosing sellar invasion. In the future, machine models can be utilized to form image reports automatically. Furthermore, the location of invasion might be accurately simulated and marked by computer vision. Machine learning can compensate for the macroscopic shortcomings of low reading efficiency and recognition bias.

In addition, the model does not have any prior medical knowledge except for two groups of images and labels. It spontaneously discovers appropriate interpretable features to assess sellar invasion. This suggests that deep learning methods can extract human-understandable domain knowledge from supervised data and have the ability to predict on the basis of the extracted knowledge.

### Strengths and Limitations

This study used the convolutional depth neural network model to identify SFI and achieve high diagnostic efficacy. After training, the model was gradually stable, and the generalization ability was excellent. This model is expected to be applied in clinical practice to assist clinicians in screening and distinguishing sellar invasion and improve reading efficiency. In addition, manual segmentation was not used in this study, which reduced the bias caused by manual factors during sampling. At the same time, this study has some limitations. Given that only contrast-enhanced sequences were collected in this study, no healthy population was used as a control group to promote the model to recognize normal sellar structures. In addition, although the model constructed in this study had good evaluation performance, the number of input images was not enough to support the model to learn more details of the sellar region. For example, the rare cases of conchal type sphenoid sinus were extremely limited in this study, the recognition ability of the model for this type was weak, and misinterpretation occurred in the testing set. Expanding the dataset and adding sequence types and healthy human saddle area images can further improve the performance and generalization ability of the model.

## Conclusion

The convolutional deep learning neural network can objectively and stably identify SFI. The CNN model has the potential to assist clinicians in accurately evaluating PA invasiveness to improve medical strategies.

## Data Availability Statement

The original contributions presented in this study are included in the article/Supplementary Material, further inquiries can be directed to the corresponding authors.

## Ethics Statement

The studies involving human participants were reviewed and approved by the Ethics Committee of the Institutional Review Board of Fuzhou 900th Hospital of Fujian Medical University and Peking Union Medical College Hospital. Written informed consent from the participants’ legal guardian/next of kin was not required to participate in this study in accordance with the national legislation and the institutional requirements.

## Author Contributions

ZL participated in the revision of the manuscript. SW and RW approved the final version to be published and contributed to reviewing the manuscript. LW carried out the statistical analyses. TF, YF, and ZP provided the medical writing and editorial support. All authors made a substantial contribution to the research design, acquisition, analysis, or interpretation of data; revised the manuscript critically; and approved the final version.

## Conflict of Interest

The authors declare that the research was conducted in the absence of any commercial or financial relationships that could be construed as a potential conflict of interest.

## Publisher’s Note

All claims expressed in this article are solely those of the authors and do not necessarily represent those of their affiliated organizations, or those of the publisher, the editors and the reviewers. Any product that may be evaluated in this article, or claim that may be made by its manufacturer, is not guaranteed or endorsed by the publisher.

## Footnotes

1
https://pytorch.org

2
https://jupyter.org
