# Supplementary figures and images for: A Convolutional Neural Network Model for Detecting Sellar Floor Destruction of Pituitary Adenoma on Magnetic Resonance Imaging Scans
Source: Front Neurosci. 2022 Jul 4;16:900519. doi: 10.3389/fnins.2022.900519 (PMC9289618; doi:10.3389/fnins.2022.900519)

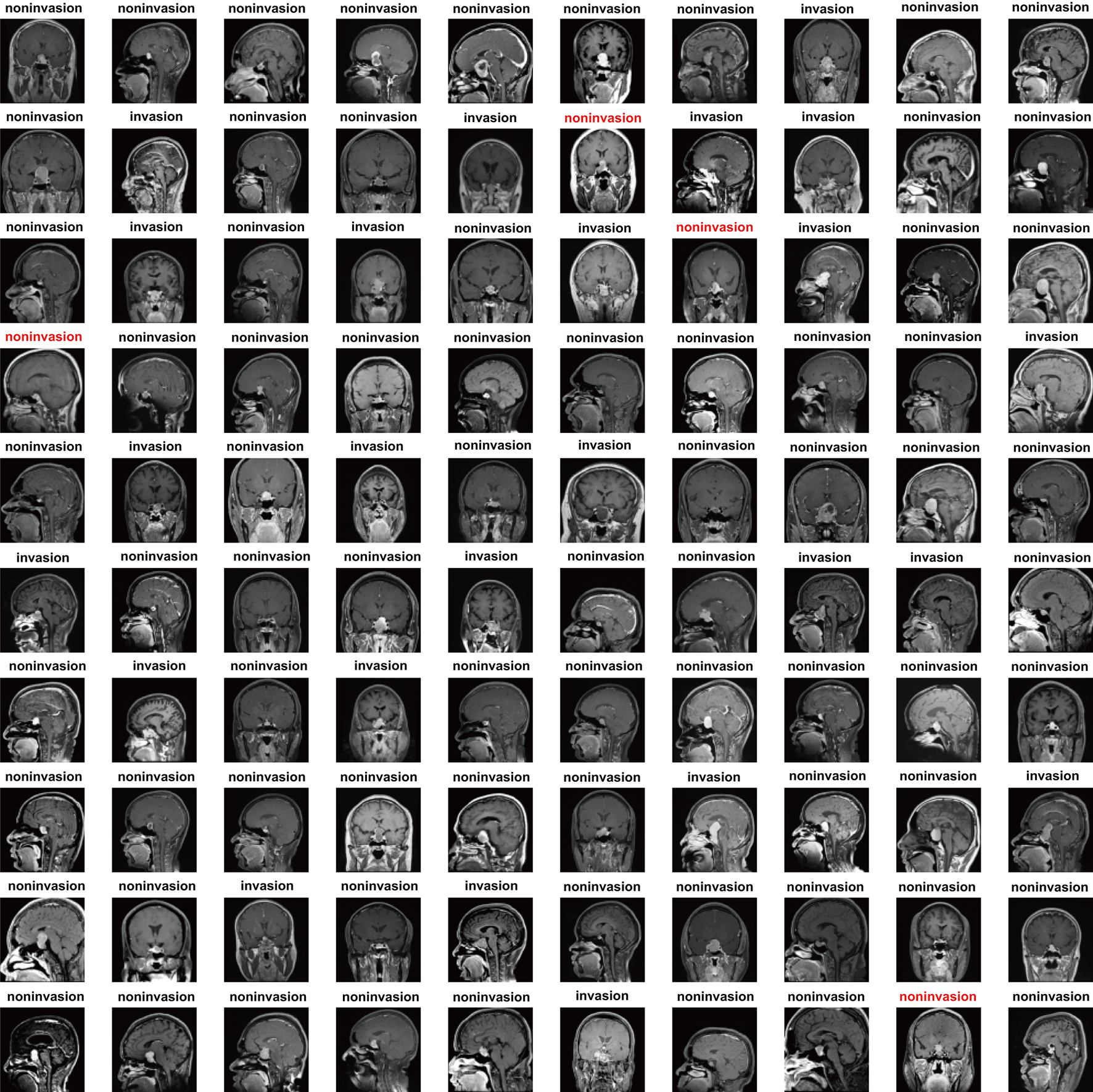

Supplement: Supplementary file 1 [file Image_1.TIF]

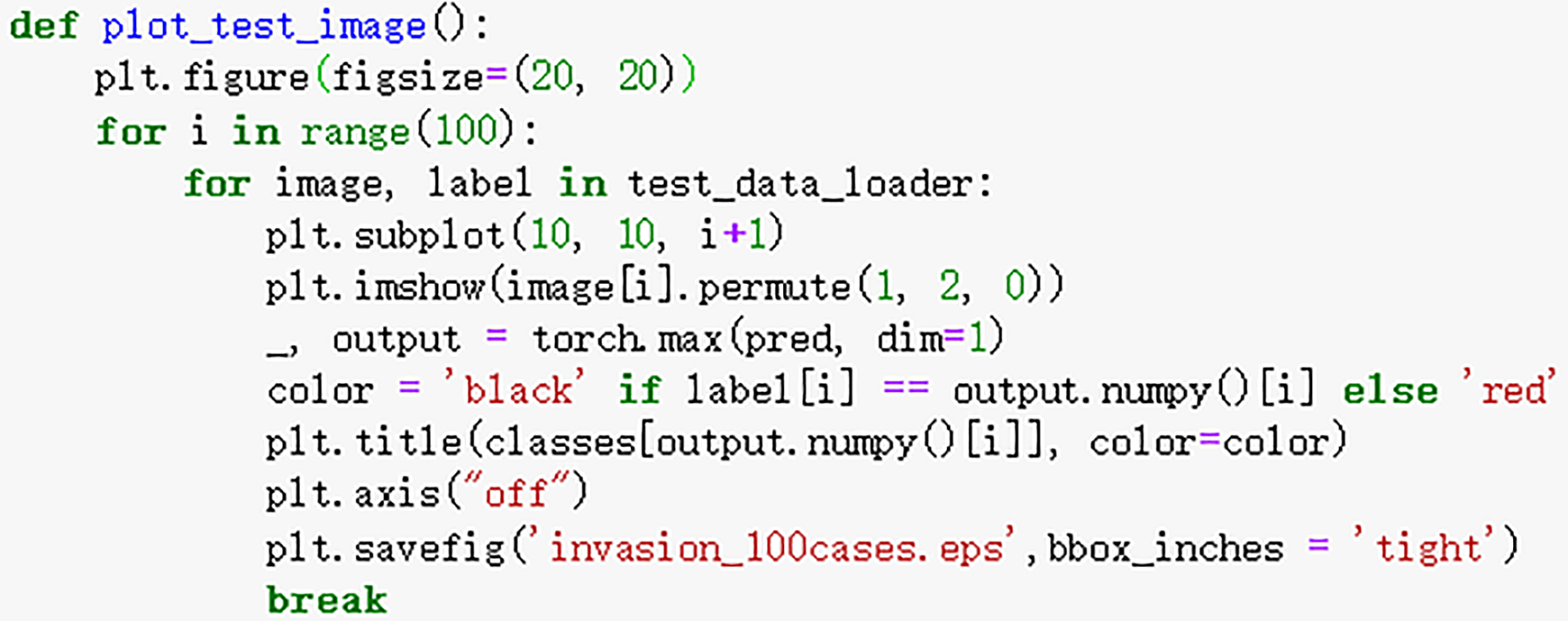

Supplement: Supplementary file 2 [file Image_2.TIF]
